# Top-down response to spatial variation in productivity and bottom-up response to temporal variation in productivity in a long-term study of desert ants

**DOI:** 10.1098/rsbl.2022.0314

**Published:** 2022-09-14

**Authors:** Heloise Gibb, Glenda M. Wardle, Aaron C. Greenville, Blair F. Grossman, Chris R. Dickman

**Affiliations:** ^1^ Department of Ecology, Environment and Evolution and Centre for Future Landscapes, La Trobe University, Melbourne, Victoria 3086, Australia; ^2^ Desert Ecology Research Group, School of Life and Environmental Sciences A08, The University of Sydney, Sydney, New South Wales 2006, Australia

**Keywords:** predator, herbivore, rainfall

## Abstract

Under the Ecosystem Exploitation Hypothesis ecosystem productivity predicts trophic complexity, but it is unclear if spatial and temporal drivers of productivity have similar impacts. Long-term studies are necessary to capture temporal impacts on trophic structure in variable ecosystems such as deserts. We sampled ants and measured plant resources in the Simpson Desert, central Australia over a 22-year period, during which rainfall varied 10-fold. We sampled dune swales (higher nutrient) and crests (lower nutrient) to account for spatial variation in productivity. We asked how temporal and spatial variation in productivity affects the abundance of ant trophic guilds. Precipitation increased vegetation cover, with the difference more pronounced on dune crests; seeding and flowering also increased with precipitation. Generalist activity increased over time, irrespective of productivity. Predators were more active in more productive (swale) habitat, i.e. spatial impacts of productivity were greatest at the highest trophic level. By contrast, herbivores (seed harvesters and sugar feeders) increased with long-term rainfall; seed harvesters also increased as seeding increased. Temporal impacts of productivity were therefore greatest for low trophic levels. Whether productivity variation leads to top-down or bottom-up structured ecosystems thus depends on the scale and dimension (spatial or temporal) of productivity.

## 1. Introduction

Ecosystem productivity drives the structure and function of ecological communities [1–4]. Greater productivity provides the resources that support more trophically complex ecosystems: a greater biomass of primary producers supports more herbivores, eventually providing the resources to support higher level consumers, including omnivores and predators [5,6] and greater specialization [7]. The relative strength of top-down and bottom-up processes varies along gradients of primary productivity, as suggested by the Exploitation Ecosystems Hypothesis (EEH, [8–10]). At low levels of productivity, primary consumers are expected to increase in abundance as primary production increases. However, at higher productivity, populations of secondary consumers will be high enough to suppress primary consumers, so only secondary consumers will appear to respond positively to productivity increases [8]. For example, herbivore density responds only weakly to increasing productivity in the presence of wolves [11].

Ecosystem productivity varies both spatially and temporally. Spatial variation in productivity can result from differences in local topography and geology because soil texture regulates water holding capacity, infiltration depth and hydraulic conductivity [12–14]. Spatial differences in plant growth, resulting from differences in water or nutrient availability, can regulate animal populations at scales that depend on their mobility [15]. Temporal fluctuations in the productivity of terrestrial ecosystems commonly result from variation in rainfall. In arid ecosystems, inter-annual variation in rainfall can be 10-fold [16,17], with dramatic impacts on net primary productivity and therefore the resources available for primary and secondary consumers [18,19]. Fluctuations in productivity can have dramatic impacts on ecosystems by structuring trophic interactions [10,20,21] and leading to switches between top-down and bottom-up control [9]. However, productivity pulses may not always be of sufficient longevity or magnitude to alter trophic structure.

In ecosystems with high temporal variation in productivity, long-term data provide critical insights into how productivity drives ecosystem structure and function. Here, we consider the response of ant assemblages to spatial and temporal variation in productivity over 22 years in the Simpson Desert in central Australia, during which rainfall varied 10-fold. We sampled ants in dune swale (higher nutrient) and crest (lower nutrient) habitats to account for spatial variation in productivity. Ants comprise a large proportion of animal biomass in many ecosystems [22] and perform important ecosystem functions [23,24]. Despite being widely regarded as omnivores, ant trophic roles range from primarily herbivorous, such as granivores, to generalists and specialized predators [25,26]. Previously, we investigated how rainfall affected the relationship between activity, species richness and dominant ants [27]. Here, we ask how responses to spatial and temporal productivity vary among ant trophic guilds. Consistent with the EEH, top-down processes are expected to be more important in driving trophic structure in more productive ecosystems as secondary consumers suppress primary consumer abundances. We therefore hypothesize that we will detect greater activity of secondary consumers (i.e. predators), in more productive landscape positions, i.e. dune swales. We expect primary consumers (i.e. herbivores) to be suppressed by secondary consumers such that we do not observe a difference in activity between dune crests and swales. By contrast, we expect that temporal increases in productivity (i.e. precipitation) will be too short-lived to allow secondary consumers to ‘catch-up' to primary consumers. Temporal changes in precipitation are therefore hypothesized to be associated with bottom-up structuring of trophic guilds, i.e. higher precipitation will drive increases in plant-based food resources and herbivores, but not predators.

## 2. Material and methods

### (a) Study site

We sampled at Ethabuka Reserve (214 000 ha), Simpson Desert, central Australia, at five sites within 10 km of ‘Main Camp' (23°46′ S, 138°28′ E). Ethabuka Reserve was a cattle station (stock density less than one animal per 100 ha [28]) until purchased by Bush Heritage Australia and destocked in 2004. The landscape is dominated by parallel sand dunes up to 10 m high and 0.6–1 km apart [29]. Each study site encompassed a crest and swale sampling point. Dune crests were open, with sparse vegetation cover including grasses, ephemeral herbaceous plants, sub-shrubs and shrubs (e.g. *Acacia ligulata*, *Dodonaea viscosa*, *Crotalaria eremaea* and *Grevillea stenobotrya*). Dune swales had heavier clay soils, up to 60% spinifex (hummock) grass (*Triodia basedowii*) cover, a similar composition of grasses, forbs and scattered shrubs and patches of Georgina gidgee (*Acacia georginae*) woodland ranging from 0.5 to 10 ha [30,31]. Swales have substantially higher moisture (approx. 3× dry mass of soils at 2 m depth and 1.2× at 20 cm depth), nitrogen (3–4×) and carbon content (6×) than crests on central Australian sand ridges [32]. Temperatures usually exceed 40°C in summer and fall below 5°C in winter [29]. Rainfall is spatially and temporally variable, and unpredictable [33]. Annual rainfall averaged 217 mm yr^−1^ (range: 79–570 mm yr^−1^) during the study period.

### (b) Invertebrate collection and traits

We sampled during the Austral spring (September, October or November) and winter (June, July or August) between 1992 and 2013 ([27]; electronic supplementary material, table S1). Ants and other invertebrates were trapped using six wet pitfall traps (40 mm diameter, 90 cm deep; filled with 3% formalin solution; left open for 2–4 consecutive days and nights) arranged in a grid of 2 × 3 (traps were separated by approx. 3 m) in crest and swale at each site. We transferred trap contents to 80% ethanol in the laboratory. Ants were counted and identified to morphospecies [34]; a reference collection was identified to species by Prof. Alan Andersen (Charles Darwin University, Darwin). We classified ants to trophic groups [35] and counts of individual ant workers per pitfall trap are reported as ant activity.

### (c) Vegetation and climate data

We sampled composition and cover of vegetation in 5 m circular plots centred on each set of six pitfall traps on most occasions that traps were set. We identified plant species and visually estimated cover to the nearest 5%. Flowering and seeding of each species were scored on a scale of 0–5, where 0 represents absence of flowering and 5 represents all individuals at peak production (detailed in [36,37]). Total plant cover, seed index (i.e. sum of seeding indices for each plot), and flowering index (sum of flowering indices for each plot) were calculated for each sampling location.

We collected climate data from the six Bureau of Meteorology weather stations closest to Main Camp: Glenormiston (104 km), Boulia (172 km), Birdsville (190 km), Marion Downs (113 km), Sandringham (65 km) and Bedourie (116 km) [38]. Monthly rainfall averaged across these stations was consistent with that averaged across local weather stations, which recorded less consistently [27]. Long-term rainfall was used to represent long-term conditions that influence colony establishment success, persistence and size (number of workers). We used cumulative rainfall in the 12 months prior to sampling [39] and short-term temperature (average minimum daily temperature in the 3 days prior to sampling) owing to its influence on ant activity [39].

### (d) Statistical analyses

**Figure 1.**
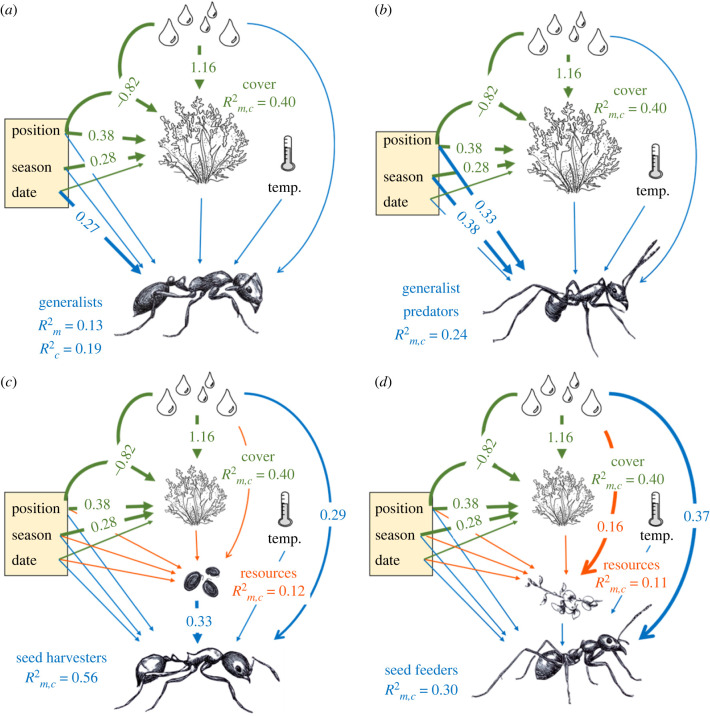
Path diagrams showing effects of precipitation, landscape position, season, date, short-term temperature and vegetation on ant trophic groups: (*a*) generalists, (*b*) generalist predators, (*c*) seed harvesters (includes seed index equation), and (*d*) sugar feeders (includes flowering equation). Thick lines indicate a significant relationship, with standardized estimates from piecewise s.e.m. shown; thin lines indicate non-significant relationships. Conditional and marginal *R*^2^ are shown for vegetation cover (‘cover', equation (2.1), shown in green), seeding index (seed harvesters) and flowering index (sugar feeders) (‘resources', equation (2.2), shown in orange) and ant trophic group (equation (2.3), shown in blue); where $R_{m}^{2}$ and $R_{c}^{2}$ were the same value this is presented as $R_{m,c}^{2}$.

We used piecewise structural equation modelling (PiecewiseSEM in R, [40]) to test how differences in productivity (both spatial and temporal) affected the activity of ant trophic groups and whether effects were direct or mediated through impacts on resources. We specified three equations: (2.1) to predict vegetation cover (green in figure 1), (2.2) (orange) to predict food resources and (2.3) (blue) to predict ant activity (electronic supplementary material, table S1):

2.1 $$\begin{array}{rl} & \mathrm{vegetation}\;\mathrm{cover}∼\mathrm{long}-\mathrm{term}\;\mathrm{precipitation}+\mathrm{season}\\ & +\mathrm{landscape}\;\mathrm{position}+\mathrm{date}\\ & +\mathrm{long}-\mathrm{term}\;\mathrm{precipitation}\times \mathrm{landscape}\;\mathrm{position}\\ & +\mathrm{site}\;(\mathrm{random}), \end{array}$$

2.2 $$\begin{array}{rl} & \mathrm{food}\;\mathrm{resources}\;(\mathrm{seeding}\;\mathrm{OR}\;\mathrm{flowering})∼\mathrm{longterm}\;\\ & \;\mathrm{precipitation}+\mathrm{vegetation}\;\mathrm{cover}+\mathrm{season}\\ & \;+\mathrm{landscape}\;\mathrm{position}+\mathrm{date}+\mathrm{site}\;(\mathrm{random}) \end{array}$$

2.3 $$\begin{array}{rl} \mathrm{and}\; & \mathrm{ant}\;\mathrm{activity}\;(\mathrm{generalists},\;\mathrm{generalist}\;\mathrm{predators},\\ & \;\mathrm{seed}\;\mathrm{harvesters}\;\mathrm{OR}\;\mathrm{sugar}\;\mathrm{feeders})\\ & \;\;\;∼\mathrm{long}-\mathrm{term}\;\mathrm{precipitation}\\ & \;\;\;\;\;\;+\mathrm{short}-\mathrm{term}\;\mathrm{temperature}+\mathrm{vegetation}\;\mathrm{cover}\\ & \;\;\;\;\;\;+\mathrm{season}+\mathrm{landscape}\;\mathrm{position}+\mathrm{date}\\ & \;\;\;\;\;\;+\mathrm{site}\;(\mathrm{random}). \end{array}$$

All three equations were used for the more herbivorous trophic groups (seeding was included for seed harvesters and flowering was used for sugar feeders in equation (2.2)) and two equations ((2.1) and (2.3)) for other trophic groups. We also included the long-term precipitation × landscape position interaction in the equation for vegetation cover, based on Akaike information criterion (AIC) values (electronic supplementary material, table S1). We did not include herbivores as food resources in equations for generalist predators as we did not expect ant predators to rely primarily on ant prey and we did not have data on other herbivores. Negative binomial response distributions were used for flowering, seeding and the activity of generalists and generalist predators; Gaussian responses were used for sugar feeder activity (double log_10_-transformed) and seed harvester activity (log_10_-transformed). All continuous predictors were scaled in the models to a mean of 0 and standard deviation of 1. No predictors in the model had a variance inflation factor greater than 2 (as recommended by Zuur *et al*. [41]). Model fit was evaluated using Fisher's C, where *p* > 0.05 indicates a good fit of the model to the data (no important paths missing).

To further test our hypothesis that effects of spatial and temporal productivity depend on trophic level, we used a generalized linear mixed model (GLMM) in the lme4 package in R [42,43] to explicitly test the trophic group : spatial productivity (position) and trophic group : temporal productivity (precipitation) interactions. We tested the model activity ∼ long-term precipitation + vegetation cover + season + landscape position + date + trophic group + trophic group : long-term precipitation + trophic group : position + site (random), using a negative binomial response. To disentangle significant interactions, we used post-hoc simple slopes analysis (for categorical : continuous interactions) and estimated marginal means tests (for categorical : categorical interactions).

From both piecewiseSEMs and the GLMM, we report marginal (fixed effects; $R_{\mathrm{GLMM}(m)}^{2}$) and conditional (fixed + random effects; $R_{\mathrm{GLMM}(c)}^{2}$.) *R*^2^ values [44] for each equation and standardized effect sizes (SES, standard deviations of the mean) for significant variables. For piecewiseSEMs, SESs for negative binomial responses were calculated using the latent theoretic approach [45].

## 3. Results

Ant activity fluctuated over time (electronic supplementary material, figure S1). Including the long-term precipitation × landscape position interaction did not improve model fit (electronic supplementary material, table S1). However, we kept this term for the equation predicting vegetation cover because it was significant, and models were within 2 AIC of the best-fit model that included no interactions.

PiecewiseSEM revealed significant effects of season, position, long-term precipitation and the interaction between long-term precipitation and position on total vegetation cover (figure 1). All models included all necessary equations: generalists: Fisher's C_4_ = 3.33, *p* = 0.193; generalist predators: Fisher's C_4_ = 3.33, *p* = 0.193; seed harvesters: Fisher's C_4_ = 8.52, *p* = 0.07; sugar feeders: Fisher's C_4_ = 4.61, *p* = 0.33. Vegetation cover was greater in spring, higher in dune swales than dune crests and increased with long-term precipitation. In periods of high rainfall, dune crests and swales had more similar vegetation cover. Flowering increased with long-term precipitation, while seeding was greater in swales than on crests (marginally non-significant).

Responses to spatial and temporal variation differed among ant trophic groups. Generalist activity increased over time, but was unaffected by other variables (figure 1*a*). Generalist predators were more active in swales than on crests and in spring than in winter (figure 1*b*). Seed harvesters increased in activity with seeding and long-term precipitation, but were similarly active on crests and in swales (figure 1*c*). Sugar feeders increased with long-term precipitation and vegetation cover (marginally non-significant), but did not respond to flowering or landscape position (figure 1*d*).

**Figure 2.**
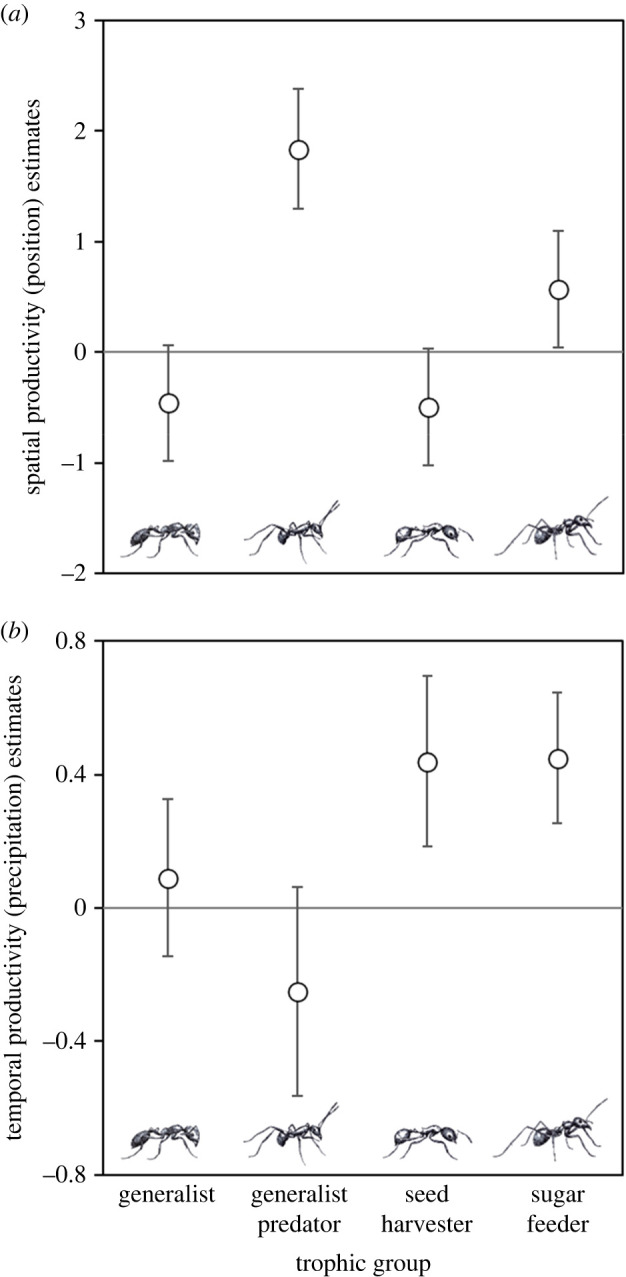
Estimates and confidence intervals for abundances of trophic groups from the post-hoc tests of interactions between: (*a*) position and trophic group (emmeans contrasts); and (*b*) trophic group and precipitation (simple slopes analysis).

Our GLMM detected significant interactions between trophic group and position and between trophic group and precipitation, consistent with our predictions (table 1) and the piecewise SEMs (figure 1). All trophic groups showed significant or near-significant effects of position on activity. However, the effect of position on predators, which were more active in the productive swale habitat, was at least three times as great as for any other trophic group (figure 2*a*). The precipitation-activity slope was significant and positive only for the more herbivorous trophic groups (seed harvesters and sugar feeders; figure 2*b*).

**Table 1.** Chi-square, significance, estimates and standard errors from the generalized linear mixed model (GLMM) testing the effect of productivity, trophic group and covariates on ant abundance. $R_{m,c}^{2}=0.69$. (Site was included as a random factor in the analysis. Significant *p*-values shown in bold. SF, sugar feeder; SH, seed harvester; G, generalist; P, generalist predator.)

source	*χ*^2^	*p*-value	estimate	s.e.
vegetation cover	1.8	0.1758	0.50	0.37
precipitation	15.8	**0.0001**	0.09	0.12
season (winter)	4.9	**0.0274**	−0.34	0.15
position (swale)	6.1	**0.0134**	0.46	0.27
trophic group	502.5	**<0.0001**	SF > SH = G > P	
date	16.1	**0.0001**	0.26	0.06
precipitation*trophic group	17.7	**0.0005**	figure 2	
position*trophic group	49.3	**0.0000**	figure 2	

## 4. Discussion

Few studies have been sufficiently long-lasting or expansive to investigate the impacts of both temporal and spatial variation in productivity on ecosystems (but see [46]). Our 22-year study of a desert ecosystem shows the importance of productivity in structuring vegetation and trophic guilds. Consistent with the EEH, secondary consumers (predators), but not primary consumers (herbivores), responded to spatial variation in productivity, indicating top-down structuring in a relatively stable productivity landscape. By contrast, temporal increases in productivity increased primary consumers, but did not lead to detectable increases in secondary consumers, suggesting that rainfall pulses were too short-lived to move the system from bottom-up to top-down structuring. We detail the impacts of productivity differences on vegetation and ant trophic groups and its broader implications.

Vegetation cover was driven by long-term precipitation, season and dune position and was thus clearly linked with water availability. Flowering, but not seeding, also increased in response to increased precipitation and the effect of increased precipitation on vegetation cover was more pronounced on dune crests than in swales, consistent with greater water limitation on dune crests.

The effects of variation in spatial and temporal productivity on ant activity differed among trophic groups, with responses linked to temporal changes in productivity for the two ‘herbivorous’ guilds and to spatial differences in productivity for predators. Both sugar feeders and seed harvesters increased in activity as precipitation increased, in agreement with studies from other systems showing that the size and activity of harvester ant colonies varies with rainfall [47]. Seed harvesters also increased with seeding. Sugar feeders responded positively to vegetation cover, but not flowering. However, floral nectar may be of minor importance compared with honeydew from insects such as psyllids, which undergo boom-bust population dynamics in response to rainfall-driven primary productivity pulses [48]. The activity of both herbivore groups thus increased as primary production increased temporally. Conversely, herbivores responded weakly to landscape position, i.e. spatial variation in productivity.

In contrast to herbivores, generalist predators increased with productivity in the spatial dimension, i.e. in the swale habitat, but did not respond to long-term precipitation. Long-lived productivity differences between dune crests and swales may cascade through to secondary consumers, allowing them to suppress herbivores, such that herbivores are not more active in higher productivity habitats. We suggest that productivity increases owing to precipitation pulses may have been too short-lived to cascade through to secondary consumers. These responses are thus consistent with expectations from the EEH [8] and previous findings that granivorous rodents respond strongly to rain-driven pulses of primary productivity, whereas mammalian generalist predators are more active in the more productive parts of the dune habitat [49]. In this study, we therefore saw a top-down response to spatial variation in productivity and bottom-up response to temporal variation in productivity.

While herbivorous and predatory ants showed clear responses to productivity, generalist ants did not. Generalist genera such as *Paraparatrechina*, *Nylanderia* and *Tapinoma* increased in activity over time, but this increase was not associated with destocking, precipitation or any vegetation variables within the timeframe of this study [27]. Over the past century, extreme rainfall events have increased in frequency and magnitude [33,50], and it is possible that the increase in generalists reflects this long-term increase in productivity. The broad diets of generalists may protect them from shorter-term fluctuations in productivity, consistent with findings that climate change creates communities dominated by generalist species [51].

In summary, our long-term study revealed stark differences in the role of spatial and temporal productivity in structuring a desert ecosystem, and greater resilience from generalists. While relatively stable spatial differences in productivity led to responses consistent with the EEH, temporal increases in productivity may have been too short-lived to induce top-down structuring. However, it is important to note that the magnitude and scale of the productivity pulse (either spatial or temporal) may be as critical as its dimension (space or time) in limiting the move from bottom-up to top-down regulation. We suggest that both the scale and dimension of productivity pulses jointly determine whether ecosystems are top-down or bottom-up regulated and look forward to further long-term research in this area.

## Data Availability

The dataset on which this article is based is available in the electronic supplementary material (data are uploaded as csv files) [52].
